# Autism-Associated Variant in the SLC6A3 Gene Alters the Oral Microbiome and Metabolism in a Murine Model

**DOI:** 10.3389/fpsyt.2021.655451

**Published:** 2021-04-15

**Authors:** Gabriella E. DiCarlo, Samuel J. Mabry, Xixi Cao, Clara McMillan, Tiffany G. Woynaroski, Fiona E. Harrison, India A. Reddy, Heinrich J. G. Matthies, Charles R. Flynn, Mark T. Wallace, Hui Wu, Aurelio Galli

**Affiliations:** ^1^Vanderbilt Brain Institute, Vanderbilt University Medical Center, Nashville, TN, United States; ^2^Department of Hearing and Speech Sciences, Vanderbilt University Medical Center, Nashville, TN, United States; ^3^Department of Surgery, University of Alabama Birmingham, Birmingham, AL, United States; ^4^Department of Neurobiology, University of Alabama Birmingham, Birmingham, AL, United States; ^5^School of Dentistry, Oregon Health and Science University, Portland, OR, United States; ^6^Department of Surgery, Vanderbilt University Medical Center, Nashville, TN, United States; ^7^Vanderbilt Kennedy Center, Vanderbilt University Medical Center, Nashville, TN, United States; ^8^Frist Center for Autism and Innovation, Vanderbilt University, Nashville, TN, United States; ^9^Department of Medicine, Vanderbilt University Medical Center, Nashville, TN, United States; ^10^Department of Psychiatry and Behavioral Sciences, Vanderbilt University Medical Center, Nashville, TN, United States

**Keywords:** autism, *Fusobacteria*, oral microbiome, dopamine transporter, mouse, metabolism

## Abstract

**Background:** Altered dopamine (DA) signaling has been associated with autism spectrum disorder (ASD), a neurodevelopmental condition estimated to impact 1 in 54 children in the United States. There is growing evidence for alterations in both gastrointestinal function and oral microbiome composition in ASD. Recent work suggests that rare variants of the SLC6A3 gene encoding the DA transporter (DAT) identified in individuals with ASD result in structural and functional changes to the DAT. One such recently identified *de novo* mutation is a threonine to methionine substitution at position 356 of the DAT (DAT T356M). The DAT T356M variant is associated with ASD-like phenotypes in mice homozygous for the mutation (DAT T356M^+/+^), including social deficits, hyperactivity, and impaired DA signaling. Here, we determine the impact of this altered DA signaling as it relates to altered oral microbiota, and metabolic and gastrointestinal dysfunction.

**Methods:** In the DAT T356M^+/+^ mouse, we determine the oral microbiota composition, metabolic function, and gastrointestinal (GI) function. We examined oral microbiota by 16S RNA sequencing. We measured metabolic function by examining glucose tolerance and we probed gastrointestinal parameters by measuring fecal dimensions and weight.

**Results:** In the DAT T356M^+/+^ mouse, we evaluate how altered DA signaling relates to metabolic dysfunction and altered oral microbiota. We demonstrate that male DAT T356M^+/+^ mice weigh less (Wild type (WT) = 26.48 ± 0.6405 g, DAT T356M^+/+^ = 24.14 ± 0.4083 g) and have decreased body fat (WT = 14.89 ± 0.6206%, DAT T356M^+/+^ = 12.72 ± 0.4160%). These mice display improved glucose handling (WT = 32.60 ± 0.3298 kcal/g, DAT T356M^+/+^ = 36.97 ± 0.4910 kcal/g), and an altered oral microbiota. We found a significant decrease in *Fusobacterium* abundance. The abundance of *Fusobacterium* was associated with improved glucose handling and decreased body fat.

**Conclusions:** Our findings provide new insights into how DAT dysfunction may alter gastrointestinal function, composition of the oral microbiota, and metabolism. Our data suggest that impaired DA signaling in ASD is associated with a number of metabolic and gastrointestinal changes which are common in individuals with ASD.

## Introduction

Autism spectrum disorder (ASD) is a neurodevelopmental condition characterized by early-emerging differences in social communication and interaction, and by patterns of restrictive and repetitive interests, behaviors, or activities (1). This condition is estimated to affect 1 in 54 children (2), with males being 4 times more likely to be identified with ASD (2), and represents an economic burden of $11.5 billion—$60.9 billion in the United States (3). There is likely no single cause of ASD. Rather, its diagnosis represents a core set of behavioral symptoms that unifies individuals with a heterogeneous collection of genetic and phenotypic differences.

Within this framework, it is critical to ascertain how the various genetic and environmental risk factors associated with ASD ultimately translate to the core symptoms of this condition and its associated comorbidities. This highlights the importance of using animals to model rare inherited variants and *de novo* mutations associated with ASD to determine their contribution to the clinical presentation of the disorder. These animal models foster the uncovering of the molecular, neurobiological, and environmental contributors to ASD.

As previously shown, features of ASD might stem from or be exacerbated by abnormal dopamine (DA) signaling (4–8). Therefore, the study of dopaminergic dysfunction as it relates to this disorder is highly relevant. Recent work from our laboratories and other investigators has identified single nucleotide polymorphisms of DAT in individuals with ASD and associated comorbidities (6–11). Among those is an ASD-associated *de novo* mutation in the *SLC6A3* gene resulting in a threonine to methionine substitution at residue 356 (DAT T356M). This mutation impairs central DA signaling and DA-dependent behaviors, promoting repetitive behaviors and hyperlocomotion that reflect behavioral characteristics seen in ASD (6, 8).

In addition to its role in central neurotransmission, DA signaling is involved in regulating functions of the enteric nervous system (ENS), a system of neurons that spans the length of the digestive system and serves to regulate digestive function (12). Dopaminergic neurons are found in the ENS and are important for gut motility, insulin release, and metabolism (13–16). Altered DA signaling promoted by the T356M mutation might not only impact central nervous system (CNS) function, but also ENS function (17). Furthermore, catecholamines, including DA, have been shown to alter the growth of some gram negative microbial species (18), including *E. coli* and *Y. enterocolitica*. Thus, changes in DA turnover (as observed in the DAT T356M^+/+^ mouse) could underlie changes in gut health, microbiota, and metabolism.

Problems with the ENS are reported to co-occur with a number of CNS disorders linked to dysregulation of the DA system. Children with ASD show multiple gastrointestinal (GI) abnormalities (19, 20). Also, they are more likely to experience abdominal pain, constipation, and diarrhea than those without ASD (21). Specifically, 30–70% of individuals with ASD have a functional GI disorder (fGID) (22). The Rome Foundation defines fGIDs as disorders of gut-brain interaction (DGBI). fGIDs are classified by GI symptoms related to any combination of motility disturbances, visceral hypersensitivity, altered gut microbiota, mucosal composition, immune function, and/or central nervous system processing (23). Evidence has suggested that these GI symptoms may exacerbate the behavioral symptoms exhibited by children with ASD by promoting emotional distress (24). Notably, maladaptive behaviors directly correlate with GI issues in individuals with ASD (25). For example, in children with ASD, behavior scores for irritability, social withdrawal, stereotypy, and hyperactivity are significantly higher in children with frequent abdominal pain, gaseousness, diarrhea, and constipation (25).

Beyond alterations in GI function, children with ASD also have an altered oral microbiota. Specifically, they have an increase in *Streptococcus* levels concomitant with a significant decrease in *Fusobacterium* (26) relative to neurotypical controls. *Fusobacterium nucleatum* is an anaerobic filamentous gram-negative bacterial species from the *Fusobacterium* genus (27, 28). It is a bacterial species implicated in a variety of infections ranging from appendicitis to osteomyelitis, as well as acting as an oncogenic bacterium implicated in colon cancer (28, 29). It is one of the most common oral bacteria found associated with a wide variety of periodontal health conditions, including gingivitis and periodontitis (30). Interestingly, *Fusobacterium* abundance has been shown to be associated with patients that display obesity and insulin resistance (31) and preterm birth (32), demonstrating a potential role of *Fusobacteria* in host metabolism.

Evidence is emerging to suggest the DA system is implicated in ASD phenotypes. Furthermore, there is ample data supporting altered gastrointestinal function and both intestinal and oral microbiome composition in ASD. To further probe this, we have generated a mouse harboring a point mutation in the SLC6A3 gene identified from a proband with ASD that displays altered DA neurotransmission (8). In this study, we explore the degree to which oral microbiome composition, metabolism, and glucose tolerance are altered by DA dysfunction in a mouse model of ASD.

## Materials and Methods

### Generation of DAT T356M^+/+^ Mouse

Mice were generated by GenOway S.A. These mice were generated and used in a previous study by the lab (8). Briefly, the point mutation was inserted into the exon 8 of the mouse SLC6A3 gene and was expressed under the control of the endogenous SLC6A3 promoter. PCR and southern blot were used to validate the proper integration of the gene. All animals used in the study were derived from matings of DAT T356M^+/−^ parents.

### Body Composition

Bruker's minispec Body Composition Analyzer was used to determine the body composition of mice based on Time Domain NMR (TD-NMR). This equipment acquires and analyzes TD-NMR signals from all protons in the entire sample volume and provides a precise method for measurement of lean tissue, fat, and free body fluid in living mice. Body composition was analyzed in mice between 14 and 19 weeks of age. Nineteen male mice and 10 female mice were utilized in these experiments.

### Caloric Expenditure

The Promethion from Sable Systems (Las Vegas, NV) was used to assess energy expenditure in 16–19 week-old mice. Ten male mice were used in these experiments. Mice were individually housed in Promethion System cages for 5 days, during which numerous parameters were continuously measured (including O_2_ consumption, CO_2_ production, food and water intake, weight, and activity). One week prior to the experiment start date, mice were singly housed for acclimation. The cages were housed in a light and temperature-controlled chamber. The light cycle was set on a 12:12 h cycle (6 am−6 pm). The temperature was maintained at 23°C for the duration of the test.

### Oral Glucose Tolerance Test

Oral glucose tolerance testing was performed in mice aged 14–20 weeks of age. 30 mice (20 males and 10 females) were used for these experiments. Animals were fasted for 4 h prior to testing. Fasted blood glucose levels were determined before a solution of 20% dextrose was administered by oral gavage. Mice were given 2 g dextrose/kg body mass. Blood glucose levels were measured at the following time points following oral gavage: 10, 20, 30, 45, 60, 75, 90, and 120 min.

### Fecal Measurements

Both male and female mice were used for these experiments. Seven mice were used in these experiments with 21 total fecal pellets measured. Mice were placed in individual transfer buckets for 5 min during the first hour of the light cycle. At the end of the 5-min period, the feces were collected. Each fecal sample was weighed using an analytical scale and measured along the longest axis using a digital caliper.

### Oral Microbiome

Sterile swabs were used to collect samples from mice oral cavity at 15–18 weeks of age. Twelve total mice were used for these experiments. Swabs were snap frozen and stored at −80°C until DNA extraction was performed.

DNA samples were extracted from swab samples with ZR Fecal/Soil DNA Miniprep Kit (Zymo Research). PCR was performed with primers specific for the V4 region of the 16S rRNA gene for amplification (33, 34). Sequences were performed on an Illumina Miseq as described previously (35, 36). Sequences were analyzed using the Quantitative Insight into Microbial Ecology (QIIME) suite v1.7 (33) and a QIIME wrapper called QWRAP (35). Operational Taxonomic Units (OTUs) were clustered at 97% sequence similarity. Taxonomic groups were assigned by using the Ribosomal Database Project (RDP) classifier (37) as well as the May 2013 Greengenes 16S rRNA sequence database (38). Samples were tested to determine whether samples clustered differently between the two groups of mice by the permutational multivariate analysis of variance (PERMANOVA) test for significant differences in clustering (*p* < 0.05). OTUs were grouped by phyla, classes, orders, families, genera and species.

### Statistics

All statistical analyses were performed using GraphPad Prism software (version 8.3.0). Data were tested for normality and homogeneity of variance when necessary. Statistical methods are indicated in the figure legends and the results section. All *t*-tests were run as two-tailed. Data are presented as mean ± SEM. Differences are considered statistically significant at *p* < 0.05.

### Ethics Approval and Consent to Participate

All behavioral and metabolic experiments were performed under a protocol approved by the Vanderbilt University Animal Care and Use Committee.

## Results

### The DAT T356M^+/+^ Mouse Has Increased Energy Expenditure

**Figure 1:**
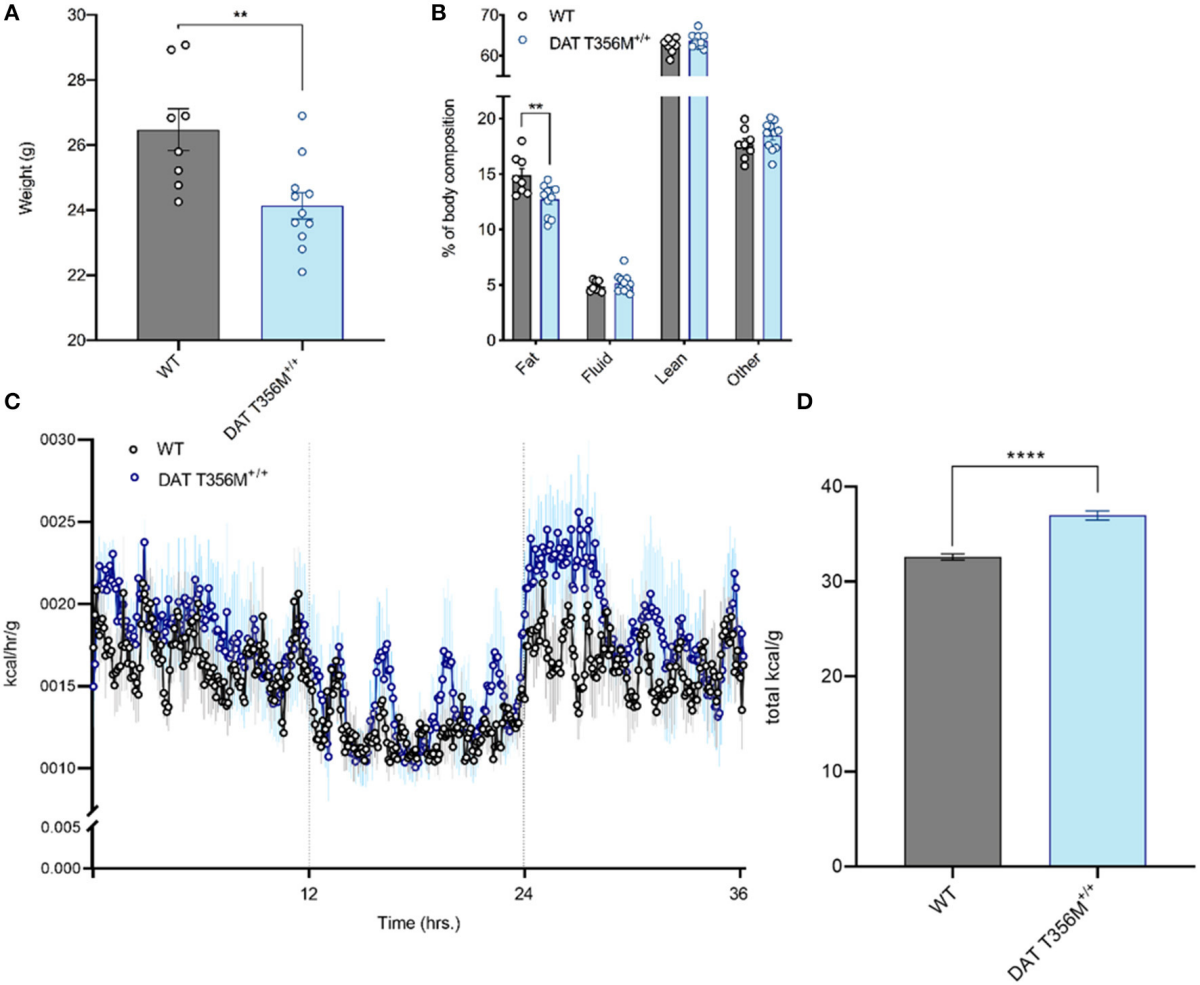
Male DAT T356M^+/+^ mice have reduced body weight, reduced percent body fat, and increased total caloric expenditure. **(A)** Total body weight is reduced in male DAT T356M^+/+^ mice (WT = 26.48 ± 0.6405 g, DAT T356M^+/+^ = 24.14 ± 0.4083 g; *n* = 8 WT, 11 DAT T356M^+/+^; *t* = 3.226, df = 17, ^**^ = *p* = 0.0050 by Student's *t*-test). **(B)** Percent body fat is reduced in male DAT T356M^+/+^ mice (WT = 14.89 ± 0.6206%, DAT T356M^+/+^ = 12.72 ± 0.4160%; *n* = 8 WT, 11 DAT T356M^+/+^; *F*_(3, 68)_ = 5.373, ^**^ = *p* = 0.0047 by two-way ANOVA followed by Sidak's multiple comparisons test). **(C)** Caloric expenditure per gram of body weight binned in 5-min intervals over the course of 36 h. Vertical lines at 12 and 24 h represent the start and end of the light cycle, respectively. The blue circles represent the mean value for DAT T356M^+/+^ mice (*n* = 5) and the black circles represents the mean value for WT mice (*n* = 5). The light gray and light blue lines represent the SEM. **(D)** Total caloric expenditure is increased in male DAT T356M^+/+^ mice as measured by indirect calorimetry (WT = 32.60 ± 0.3298 kcal/g, DAT T356M^+/+^ = 36.97 ± 0.4910 kcal/g, *n* = 5 WT, 5 DAT T356M^+/+^, *t* = 7.388, df = 8, ^****^ = *p* < 0.0001 by Student's *t*-test).

We have previously shown that the DAT T356M^+/+^ mouse has impaired central DA neurotransmission (8). Since DA is implicated in regulating metabolism, most notably in insulin release (14–16), and ENS function (13), we first measured body weight and body composition of DAT T356M^+/+^ mice and their wild type (WT) littermates. Body weight was significantly reduced in male DAT T356M^+/+^ mice compared to male WT mice at 14–19 weeks of age (Figure 1A. WT = 26.48 ± 0.6405 g, DAT T356M^+/+^ = 24.14 ± 0.4083 g; *n* = 8 WT, 11 DAT T356M^+/+^; *t* = 3.226, df = 17, *p* = 0.0050 by Student's *t*-test). However, there was no difference in body weight between female DAT T356M^+/+^ and WT mice (WT = 22.78 ± 0.8938 g, DAT T356M^+/+^ = 22.18 ± 0.7658 g; *n* = 4 WT, 6 DAT T356M^+/+^; *t* = 0.5015, df = 8, *p* = 0.6296 by Student's *t*-test), suggesting that this effect is sexually dimorphic.

To better understand how the T356M mutation influences mouse body weight, we measured body composition of male and female DAT T356M^+/+^ mice and WT mice. In males, we found significantly reduced percent body fat in DAT T356M^+/+^ mice compared with WT mice (Figure 1B. WT = 14.89 ± 0.6206%, DAT T356M^+/+^ = 12.72 ± 0.4160%; *n* = 8 WT, 11 DAT T356M^+/+^; *F*_(3, 68)_ = 5.373, *p* = 0.0047 by two-way ANOVA followed by Sidak's multiple comparisons test). No other components of the body composition (i.e., lean mass, fluid, or other) were different between male DAT T356M^+/+^ mice and WT mice (Figure 1B). In contrast, no differences in body composition were observed between female DAT T356M^+/+^ mice and WT mice (Body fat: WT = 13.23 ± 0.5502%, DAT T356M^+/+^ = 13.17 ± 0.2128%, Fluid: WT = 5.987 ± 0.1256%, DAT T356M^+/+^ = 5.752 ± 0.3474%, Lean: WT = 64.17 ± 0.776*%*, DAT T356M^+/+^ = 63.15 ± 0.3714%; *n* = 4 WT, 6 DAT T356M^+/+^, *F*_(1, 24)_ = 1.631 and *p* = 0.2137 for genotype, *F*_(2, 24)_ = 0.7398 and *p* = 0.4878 for interaction between genotype and tissue type by two-way ANOVA). In male DAT T356M^+/+^ mice, this decrease in body weight is driven, at least in part, by a decrease in percent body fat and not lean mass (Figure 1B). Exercise can regulate body composition (i.e., decreased body fat and increased lean mass) (39).

In 7 week old heterozygous animals (DAT T356M^+/−^) we did not observe differences in body weight with respect to WT. To note, is that these animals were younger than the animals used in other experiments in this study. This cohort was made of entirely male mice and there was no significant difference in weight between WT and DAT T356M^+/−^ mice (WT = 21.42 ± 3.57 g, DAT T356M^+/−^ = 20.85 ± 2.75g, *n* = 13 WT, 11 DAT T356M^+/−^, *t* = 0.776, df = 22, *p* = 0.446 by Students *t*-test).

We used a metabolic cage (Promethion, Sable Systems, Las Vegas, USA) to measure energy expenditure continuously over 36 h both in male DAT T356M^+/+^ mice and WT mice (Figure 1C). Total energy expenditure over 36 h was significantly increased in male DAT T356M^+/+^ mice (Figure 1D. WT = 32.60 ± 0.3298 kcal/g, DAT T356M^+/+^ = 36.97 ± 0.4910 kcal/g, *n* = 5 WT, 5 DAT T356M^+/+^, *t* = 7.388, df = 8, *p* < 0.0001 by Student's *t*-test). As the DAT T356M^+/+^ mice are hyperactive (8), one would expect lean mass to be increased. However, since we saw no change in lean mass our data indicate that this decrease in body mass is not likely promoted by hyperactivity, but rather by metabolic changes.

### DAT T356M^+/+^ Mice Have Improved Glucose Tolerance

It is well-established that DA also plays an important role in glucose and insulin regulation (16, 40, 41). Insulin-secreting pancreatic β-cells express the enzymes required for DA synthesis and catabolism, as well as all five DA receptors (40, 42). In these cells, DA functions as a negative regulator of glucose-stimulated insulin secretion (GSIS) (41). The D2 receptor (D2R) and D3 receptor (D3R) signaling act in concert to inhibit GSIS (14–16). Consistent with these findings, β-cell-selective D2R knockout mice exhibit marked postprandial hyperinsulinemia (16).

**Figure 2:**
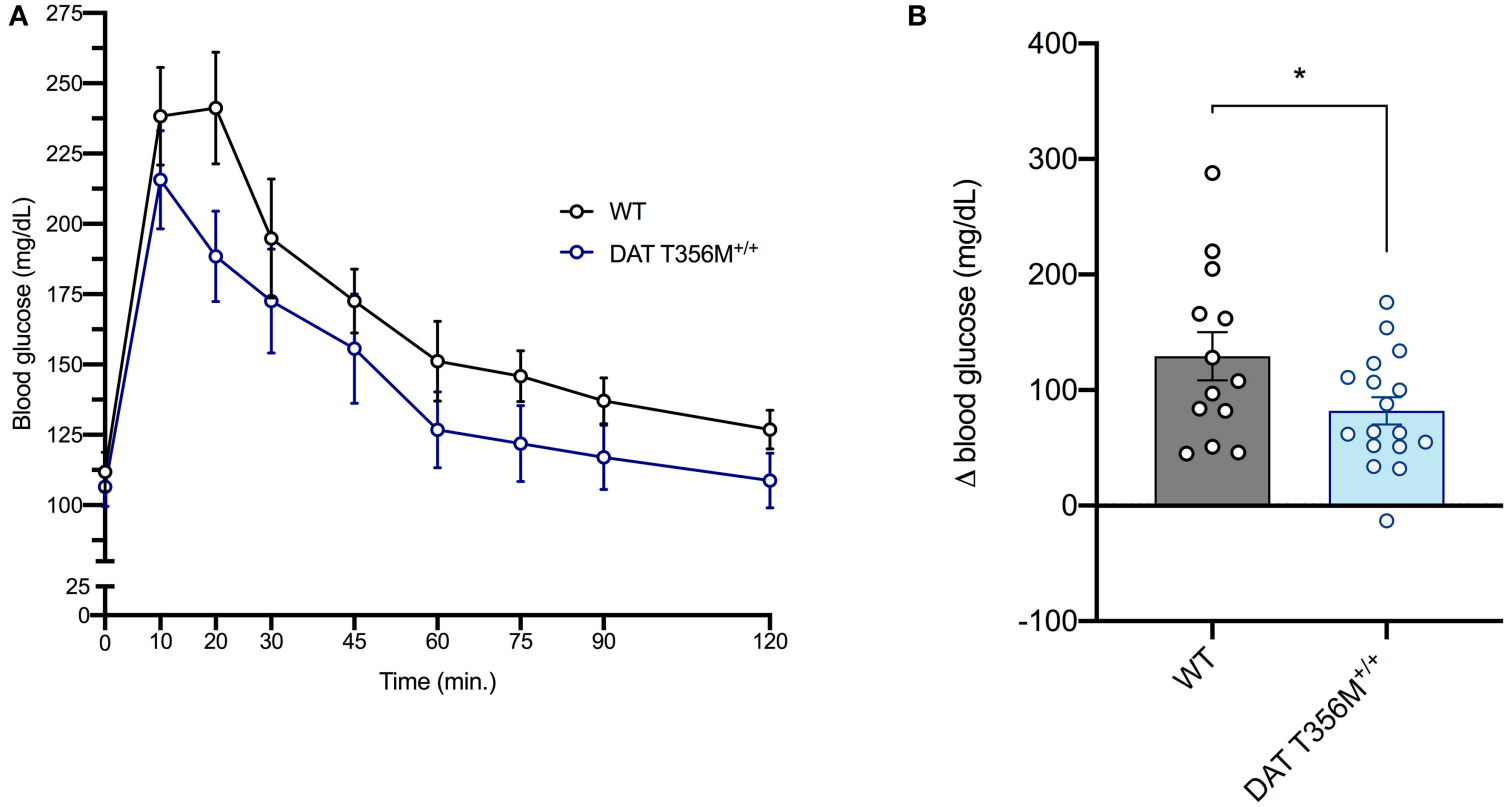
DAT T356M^+/+^ mice have improved glucose handling after glucose challenge. **(A)** Blood glucose levels during a glucose tolerance test in WT and DAT T356M^+/+^ mice. **(B)** Change in blood glucose from baseline to 20 min (Δ blood glucose) is significantly reduced in DAT T356M^+/+^ mice compared to WT mice (WT = 129.4 ± 20.80 mg/dL, DAT T356M^+/+^ = 81.94 ± 11.78 mg/dL; *n* = 13 WT, 17 DAT T356M^+/+^; *t* = 2.100, df = 28, ^*^ = *p* = 0.0448 by Student's *t*-test).

DAT T356M^+/+^ mice have impaired D2 receptor signaling (8). Thus, we sought to determine whether glucose homeostasis was also affected in these animals. After glucose loading, there was a rapid decrease in blood glucose in DAT T356M^+/+^ mice, while this rapid decrease in blood glucose is not present in WT mice (Figure 2A). Maximum blood glucose levels were lower in DAT T356M^+/+^ mice than WTs and returned to baseline faster than WTs, which still had slightly elevated levels even after 120 min. These results highlight that there is significantly improved glucose handling in the DAT T356M^+/+^ mice (Figure 2B. WT = 129.4 ± 20.80 mg/dL, DAT T356M^+/+^ = 81.94 ± 11.78 mg/dL; *n* = 13 WT, 17 DAT T356M^+/+^; *t* = 2.100, df = 28, *p* = 0.0448 by Student's *t*-test).

### The DAT T356M^+/+^ Mouse Displays Reduced Abundance of Oral *Fusobacterium*

**Figure 3:**
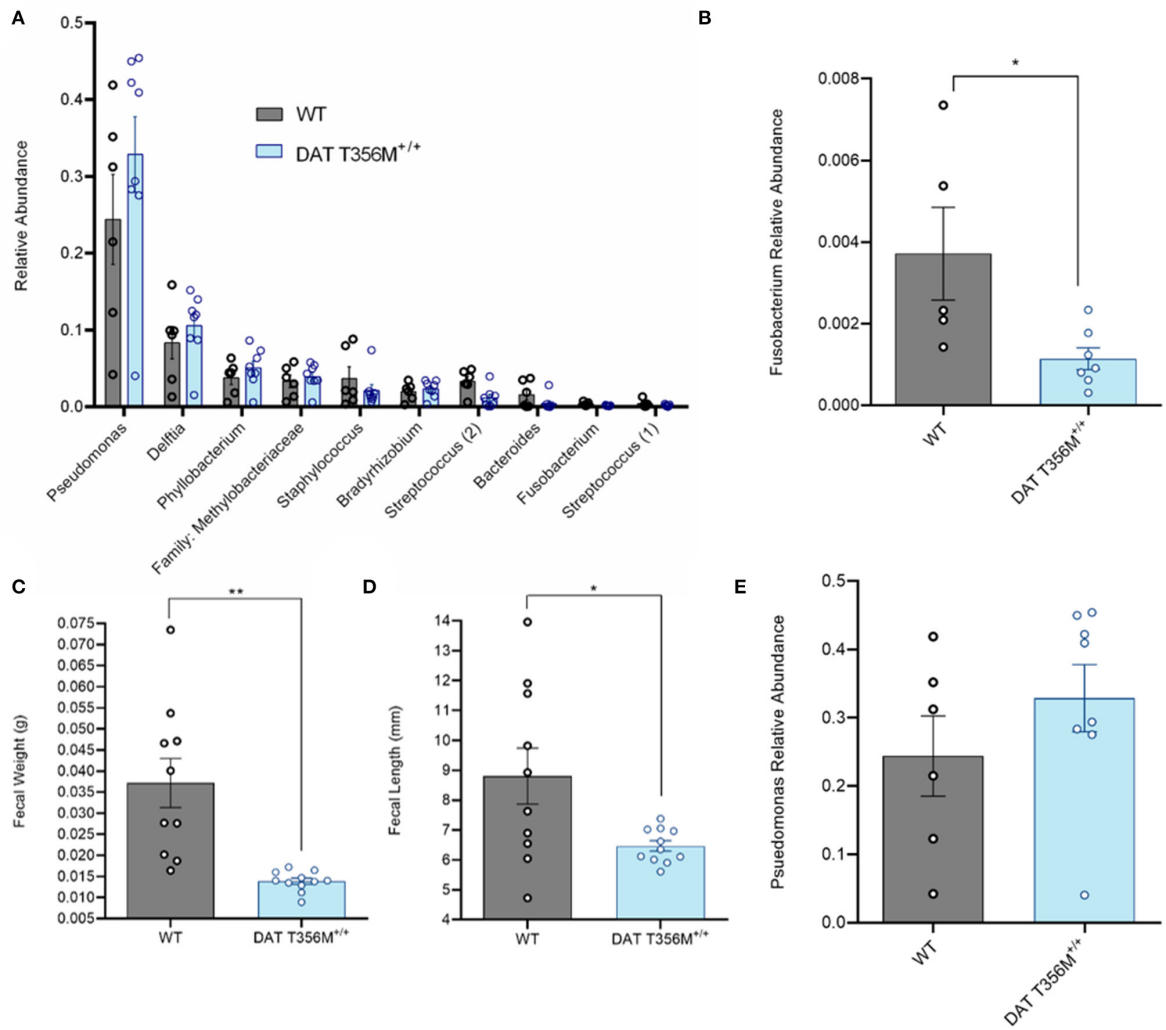
DAT T356M^+/+^ mice have altered oral microbiota. **(A)** Relative abundance of top 10 OTUs isolated from oral swabs from WT and DAT T356M^+/+^ mice. **(B)** DAT T356M^+/+^ mice have significantly lower abundance of oral *Fusobacterium* compared to WT mice (WT = 0.003716 ± 0.001136 relative units, DAT T356M^+/+^ = 0.001 ± 0.0003 relative units; *n* = 5 WT, 7 DAT T356M^+/+^; *t* = 2.589, df = 10, * = *p* = 0.027 by Student's *t*-test). **(C)** DAT T356M^+/+^ mice have significantly decreased fecal weight compared to WT mice (WT = 0.0371 ± 0.0058 g, DAT T356M^+/+^ = 0.0138 ± 0.0139 g; *n* = 10 fecal pellets from 3 WT mice, 11 fecal pellets from 4 DAT T356M^+/+^ mice; *t* = 3.981, df = 9, ** = *p* = 0.0032 by Welch's *t*-test). **(D)** DAT T356M^+/+^ mice have significantly decreased fecal length compared to WT mice (WT = 8.805 ± 0.9381 mm, DAT T356M^+/+^ = 6.469 ± 0.1727 mm; *n* = 10 feces from 3 WT mice, 11 feces from 4 DAT T356M^+/+^ mice; *t* = 2.449, df = 9, * = *p* = 0.0368 by Welch's *t*-test). **(E)** There is no difference in the relative abundance of *Pseudomonas* between WT and DAT T356M^+/+^ mice (WT = 0.2438 ± 0.05866 relative units, DAT T356M^+/+^ = 0.3286 ± 0.0491 relative units; *n* = 6 WT, 8 DAT T356M^+/+^; *t* = 1.113, df = 12, *p* = 0.2873 by Students *t*-test).

A number of changes in gut and oral microbial populations have been reported in individuals with ASD (26, 43). Strong evidence points to a decrease in the abundance of oral *Fusobacterium* in persons with ASD (26). Changes in these bacterial populations have also been associated with alterations in various metabolic processes. For example, increased *Fusobacterium* in the gut correlates with insulin resistance (31). Thus, we sought to determine the composition of the oral microbiota and, specifically, the abundance of *Fusobacterium* in DAT T356M^+/+^ mice compared to WT mice. In Figure 3A, we show the top 10 operational taxonomic units (OTUs) isolated from oral swabs from DAT T356M^+/+^ and WT mice. We found that *Fusobacterium* was significantly decreased in the DAT T356M^+/+^ mice (Figure 3B. WT = 0.003716 ± 0.001136 relative units, DAT T356M^+/+^ = 0.001 ± 0.0003 relative units; *n* = 5 WT, 7 DAT T356M^+/+^; *t* = 2.589, df = 10, *p* = 0.027 by Student's *t*-test). However, the decrease in the abundance seen in *Fusobacterium* is not observed for other bacteria, such as *Pseudomonas*, the most abundant OTU isolated from our samples (Figures 3A,E. WT = 0.2438 ± 0.05866 relative units, DAT T356M^+/+^ = 0.329 ± 0.0491 relative units; *n* = 6 WT, 8 DAT T356M^+/+^; *t* = 1.113, df = 12, *p* = 0.2873 by Student's *t*-test).

To evaluate the impact of the DAT T356M on GI function, we analyzed fecal weight and length. In a mouse model of constipation, there is a decrease in both fecal weight and length (44), demonstrating that changes in these parameters can be used as a partial readout of GI function. DAT T356M^+/+^ mice displayed reduced fecal weight (Figure 3C. WT = 0.037 ± 0.0058 g, DAT T356M^+/+^ = 0.01388 ± 0.01388 g; *n* = 10 from 3 WT, *t* = 3.981, df = 9, 11 from 4 DAT T356M^+/+^; *p* = 0.0032 by Welch's *t*-test) and length (Figure 3D. WT = 8.805 ± 0.9381 mm, DAT T356M^+/+^ = 6.469 ± 0.1727 mm; *n* = 10 from 3 WT, 11 from 4 DAT T356M^+/+^; *t* = 2.449, df = 9, *p* = 0.0368 by Welch's t-test), suggesting constipation in DAT T356M^+/+^ mice (44).

### Relative Abundance of Oral *Fusobacterium* Is Positively Correlated With Percent Body Fat and Peak Blood Glucose

**Figure 4:**
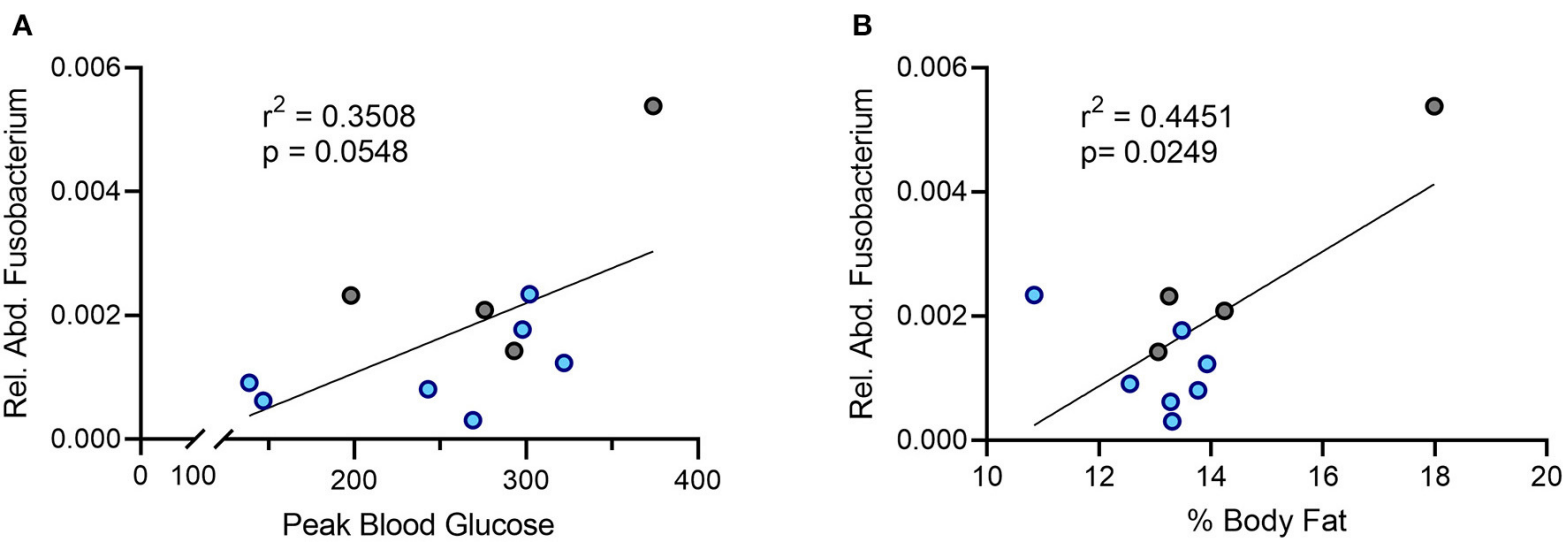
There is a positive correlation between the relative abundance of Fusobacterium and both peak blood glucose and percent body fat. **(A)** Peak blood glucose is positively correlated with relative abundance of oral *Fusobacterium* (Pearson's *r* = 0.5923; *n* = 4 WT, 7 DAT T356M^+/+^; *p* = 0.058). **(B)** The relative abundance of *Fusobacterium* in the oral cavity and peak blood glucose are positively and significantly correlated (Pearson's *r* = 0.6671; *n* = 4 WT, 7 DAT T356M^+/+^; *p* = 0.0249). Blue dots represent T356M^+/+^ mice and gray dots represent WT mice.

In humans, an increased abundance of *Fusobacterium* correlates with insulin resistance (31). Given the improved glucose handling in the DAT T356M^+/+^ mice, we sought to determine the relationship between relative abundance of oral *Fusobacterium* and metabolic parameters in our sample. We observed a positive association between the relative abundance of *Fusobacterium* in the oral cavity and peak blood glucose during a glucose challenge (Figure 4A, Pearson's *r* = 0.5923; *n* = 4 WT, 7 DAT T356M^+/+^; *p* = 0.058). Although this correlation did not reach statistical significance, it was notably strong in magnitude. We similarly observed a strong, positive and significant association between the relative abundance of *Fusobacterium* in the oral cavity and percent body fat (Figure 4B, Pearson's *r* = 0.6671; *n* = 4 WT, 7 DAT T356M^+/+^; *p* = 0.0249). To determine aforementioned relations were limited to *Fusobacterium*, we additionally analyzed the associations for relative abundance of *Pseudomonas* with both percent body fat and peak blood glucose. We found no significant correlation between relative abundance of *Pseudomonas* and either percent body fat (Pearson's *r* = 0.1208; *n* = 6 WT, 8 DAT T356M^+/+^; *p* = 0.2233) or peak blood glucose (Pearson's *r* = 9.7 × 10^−6^; *n* = 6 WT, 8 DAT T356M^+/+^; *p* = 0.99). These correlations were negligible to small in magnitude.

## Discussion

The mechanistic understanding of proteins, such as DAT, that regulate DA neurotransmission is vital to pharmacologically target DA dysfunction in autism and other conditions. DAT variants, dysregulation of DA, and altered developmental trajectory of dopaminergic structures have been associated with ASD (6, 9–11, 45, 46). Beyond the importance of proper DA neurotransmission for central function, increasing evidence highlights the role of DA signaling in gut motility, insulin release, and metabolism. In the pancreas, DA is stored in secretory granules in pancreatic beta-islet cells and regulates GSIS (14, 15). Moreover, in the intestines, dopaminergic neurons are found in the ENS and are important for proper intestinal motility (13). As such, it is not surprising that dysfunction in the DA system can affect both metabolism and GI function. Thus, studying DAT variants associated with ASD will facilitate understanding of their role in central and peripheral neurotransmitter homeostasis and will help to determine how transporter dysfunction contributes to GI and metabolic disorders.

Here we explore the impact of an ASD-associated *de novo* mutation of the DAT (6, 8), which impacts DA neurotransmission, on metabolism, glucose handling, and the oral microbiome. We demonstrate that DAT T356M^+/+^ mice exhibit altered body composition, energy expenditure, glucose handling, and composition of oral microbiota. This study links altered dopaminergic signaling due to DAT dysfunction caused by an ASD-associated genetic variant with pathophysiological changes that recapitulate aspects of the human disorder in a murine model.

We first explored the impact of the DAT T356M mutation on energy homeostasis. We found that male DAT T356M^+/+^ mice have reduced body weight and that this reduction in body weight is characterized predominantly by reduced body fat, without a significant change in fluid, lean, or other body tissues. Interestingly, no difference in body composition were observed in female mice. Males have been shown to have sex specific deficits in other animal models of autism (47–49). Thus, a more pronounced phenotype in our male mice is in line with previous data. Since no change in lean mass was observed, it is unlikely that this change in body fat is driven by hyperactivity. Rather, this reduction in body fat is more likely associated with increased total energy expenditure.

As DA signaling regulates insulin release (16), we additionally asked whether the observed reduction in body fat was associated with altered glucose handling. We found that DAT T356M^+/+^ mice display significant differences in glucose handling (specifically, a significantly lower change in blood glucose 20 min after glucose challenge in male mice). Furthermore, in pancreatic beta-islet cells, the D2 autoreceptor negatively regulates the release of insulin (14, 16). Considering that DAT T356M^+/+^ mice have reduced D2 receptor signaling (8), we therefore suggest that this improvement in glucose handling may be due to increased insulin release. Furthermore, it is important to note that, when compared to skeletal muscle, adipose tissue is not generally thought as being a major user of enteral (i.e., gastrointestinal) glucose. Therefore, we suggest that this altered glucose handling in the DAT T356M^+/+^ mice is likely not due to their lowered fat composition, but rather changes in the sensitivity of the D2 receptor. Future studies should directly assess the insulin levels seen in these mice, as well as the sensitivity and expression of D2 receptor in beta-islet cells in the pancreas to determine the underlying physiology of the improved glucose handling seen in our animal model.

In addition to insulin release, DA plays a critical role in GI function (13, 17). Children with ASD display multiple GI abnormalities (19, 20) with 30–70% of individuals with ASD having a fGID (22), a group of disorders classified by motility disturbances, altered mucosal function, and altered gut microbiota (23). As a read out for proper GI function, we measured both the length and weight of feces in DAT T356M^+/+^ mice and discovered that their feces are lighter and shorter than WT mice. These alterations have been previously observed in mouse models of constipation (44). Notably, children with ASD are more likely to experience abdominal pain and constipation when compared to those without ASD (21).

Many studies have reported differences in the composition of the oral and gut microbiota in patients with ASD (26, 50) and it is possible that these differences in the flora may contribute to both behavioral and GI symptoms associated with autism (21, 24). As communication between the brain and the gut is bidirectional, it is conceivable that dysfunction of key regulators of CNS function associated with ASD may also drive dysfunction of the ENS and thus changes in the composition of the oral and GI microbiota (51, 52). Here, we point to the DAT as one such key regulator. We found that DAT T356M^+/+^ mice have reduced abundance of *Fusobacterium* in the oral cavity and no difference in *Pseudomonas*, the most abundant bacteria in the oral cavity of our animals, as compared to WT mice. It is possible that this change in oral microbiome is due directly to the anomalous DA release seen in this mutant (8), as there is evidence that DA can alter the growth of certain gram-negative bacterial species (18). However, we cannot rule out the possibility that this change is due to dietary differences or differences in metabolism leading to an altered microbiome. Consistent with our data, increased *Fusobacterium* abundance has been associated with insulin resistance and impaired glucose handling in obese patients (31). Of note, we found a positive association between the relative abundance of *Fusobacterium* and both peak blood glucose and percent body fat in our mouse model. However, there was no relation between the abundance of *Pseudomonas* and either peak blood glucose or percent body fat.

Our data demonstrate that there is less *Fusobacterium* in the oral microbiome in the DAT T356M^+/+^ model of ASD, a finding that is in agreement with human data from Qiao and colleagues (26). *Fusobacterium nucleatum* (*F. nucleatum*) is one of the most common species in periodontal diseases, such as gingivitis (30). These findings, collectively, point toward a potential explanation for the fact that, despite having no difference in quality of teeth brushing (53), children with ASD have been observed to display better oral health and less caries when compared to a control population (53, 54). Given that both humans and our murine model display significantly less oral *Fusobacterium*, this could potentially be a driving factor in the superior oral hygiene seen in children with ASD, despite no differences in teeth brushing habits (53). Consistent with our hypothesis, a recent study from Schoilew and colleagues demonstrated that patients without a history of caries have significantly less *Fusobacterium* than patients that previously had caries (55). Alternatively the reduction in *F. nucleatum* level may mediate some of the phenotypes seen in ASD as *F. nucleatum* is known to produce butyrate and this is a short chain fatty acid that has been shown to be present at decreased levels in individuals with ASD (56). It is speculated that the alterations in the microbiome and the decrease in butyrate is one of the drivers of the constipation and other GI issues observed in ASD (56). Clearly, this observation requires more research on both human patients and alternative animal models of ASD to corroborate differences presented in other studies and explore underlying mechanisms (57).

Here, we present new evidence of how DAT dysfunction may translate to altered GI function, composition of the microbiota of the oral cavity, and metabolism. This model suggests that impaired DA signaling in ASD drives a number of pathophysiological changes that could explain, in part, at least some of the observed metabolic and GI phenotypes in ASD. This work also suggests that DA signaling, or specific microbial populations in the microbiome, may represent a tractable target for the treatment of GI and metabolic disturbances in persons effected by ASD, and potentially in individuals with other conditions associated with DA dysfunction.

## Data Availability Statement

The datasets presented in this study can be found in online repositories. The names of the repository/repositories and accession number(s) can be found in the article/Supplementary Material.

## Ethics Statement

The animal study was reviewed and approved by Vanderbilt University Animal Care and Use Committee.

## Author Contributions

GD, SM, XC, and CM designed and performed experiment and contributed to the writing of the manuscript. TW, FH, IR, HM, CF, MW, HW, and AG designed the experiments and contributed to writing the manuscript. All authors contributed to the article and approved the submitted version.

## Conflict of Interest

The authors declare that the research was conducted in the absence of any commercial or financial relationships that could be construed as a potential conflict of interest.

## Abbreviations

DA: dopamine
DAT: dopamine transporter
WT: wild type
ASD: autism spectrum disorder
CNS: central nervous system
ENS: enteric nervous system
GI: gastrointestinal
fGID: functional GI disorder
DGBI: disorder of gut brain interaction.
