# Supplementary material for: Autism-Associated Variant in the SLC6A3 Gene Alters the Oral Microbiome and Metabolism in a Murine Model
Source: Front Psychiatry. 2021 Apr 15;12:655451. doi: 10.3389/fpsyt.2021.655451 (PMC8081952; doi:10.3389/fpsyt.2021.655451)
Supplement: Supplementary file 1 [file Data_Sheet_1.DOCX]

The accession numbers are below (I submitted 15 different samples, one for each animal that who had their oral microbiome sequenced for the manuscript).

18235460: <https://www.ncbi.nlm.nih.gov/sra/18235460>

18235461: <https://www.ncbi.nlm.nih.gov/sra/18235461>

18235462: <https://www.ncbi.nlm.nih.gov/sra/18235462>

18235463: <https://www.ncbi.nlm.nih.gov/sra/18235463>

18235464: <https://www.ncbi.nlm.nih.gov/sra/18235464>

18235465: <https://www.ncbi.nlm.nih.gov/sra/18235465>

18235466: <https://www.ncbi.nlm.nih.gov/sra/18235466>

18235467: <https://www.ncbi.nlm.nih.gov/sra/18235467>

18235468: <https://www.ncbi.nlm.nih.gov/sra/18235468>

18235469: <https://www.ncbi.nlm.nih.gov/sra/18235469>

18235470: <https://www.ncbi.nlm.nih.gov/sra/18235470>

18235471: <https://www.ncbi.nlm.nih.gov/sra/18235471>

18235472: <https://www.ncbi.nlm.nih.gov/sra/18235472>

18235473: <https://www.ncbi.nlm.nih.gov/sra/18235473>

18235474: [https://www.ncbi.nlm.nih.gov/sra/1823547](https://www.ncbi.nlm.nih.gov/sra/18235474)4
